# A 3D MoS_2_/Graphene Microsphere Coated Separator for Excellent Performance Li-S Batteries

**DOI:** 10.3390/ma11102064

**Published:** 2018-10-22

**Authors:** Shuang Yang, Junfan Zhang, Taizhe Tan, Yan Zhao, Ning Liu, Haipeng Li

**Affiliations:** 1 School of Materials Science and Engineering, Hebei University of Technology, Tianjin 300130, China; 15522560785@163.com (S.Y.); 18722593259@163.com (J.Z.); 2 Synergy Innovation Institute of GDUT, Heyuan 517000, China; tztansii18@163.com

**Keywords:** Li-S battery, shuttle effect, MoS_2_/graphene-coated separator

## Abstract

Lithium-sulfur (Li-S) batteries are the most prospective energy storage devices. Nevertheless, the poor conductivity of sulfur and the shuttling phenomenon of polysulfides hinder its application. In this paper, flower-like MoS_2_/graphene nanocomposite is prepared and deposited on a multi-functional separator to enhance the electrochemical behavior of Li-S batteries. The results demonstrated that the MoS_2_/graphene-coated separator is contributing to inhibit the shuttling phenomenon of polysulfides and improve the integrity of sulfur electrode. The initial discharge capacity of the battery using MoS_2_/graphene-coated separator at 0.2 C was up to 1516 mAh g^−1^. After 100 cycles, a reversible capacity of 880 mAh g^−1^ and a coulombic efficiency of 98.7% were obtained. The improved electrochemical behavior can be due to the nanostructure and Mo-S bond of the MoS_2_/graphene composite, which can combine physical shielding and chemisorption to prohibit the shuttle effect of polysulfides. The results prove that the MoS_2_/graphene-coated separator has the potential for feasible application in Li-S batteries to enhance their electrochemical performance.

## 1. Introduction

Lithium-sulfur (Li-S) battery exhibits enormous potential to be the next generation of low-cost and high-capacity batteries due to their high theoretical capacity (1675 mAh g^−1^), abundance reserves and eco-friendly nature [1,2]. With these merits, the commercial application of Li-S batteries, however, is still hindered due to the shuttling behavior of reduction compounds, the poor conductivities of sulfur, and the huge volume changes of the sulfur electrode in charge/discharge processes [3,4,5]. Among these deficiencies, the dissolution and diffusion of lithium polysulfides (LPS), which leads to the decrease of active materials utilization, capacity fading and self-discharge, seems to be the most challenging problem. Thus, the key point to develop Li-S batteries with excellent performance is preventing LPS shuttling in the electrolyte.

Multifarious strategies have been used to tackle this issue, such as integration of a conductive framework with sulfur [6,7,8,9,10], and optimization of the organic electrolyte [11,12]. Recently, much attention has been directed to incorporation of functional interlayers or multifunctional separators. The separator is used as an ionic conductor and electronic insulator in battery systems. Moreover, the electrolyte as well as polysulfides can pass through the separator and diffuse between the cathode and anode during the charge/discharge processes [13]. Multifunctional separators combine the barrier layer with the adsorption function layer into an integration structure, resulting in a superior performance than functional interlayers [14,15,16,17]. In addition, multi-functional separators offer the advantages of being inexpensive, of having high yield and also the viability of production on an industrial-scale. To date, there are several types of multi-functional separators, including carbon-modified separator [18,19], polymer-modified separator [20,21], oxide-modified separator [22], and metal sulfides-modified separator [23], that have been used in Li/S batteries. Compared with other modified methods, metal sulfides-modified separator has attracted more research attention because that metal–S bonds of metal sulfides do not only adsorb polysulfides dipolar via interaction on the polarized surface, but also, restrict polysulfides through the stronger S–S interaction. The previous literature has reported that the dissolution/diffusion of LPS can be significantly debased by the strong chemical bonding of sulfur to the metal sulfides, and the batteries exhibit an excellent capacity [24,25].

**Figure 1:**
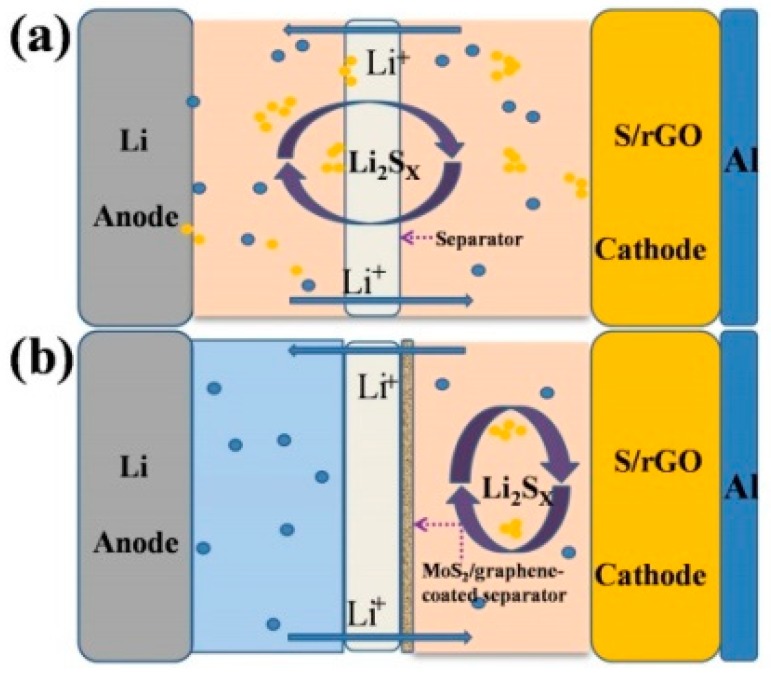
The schematic illustration of polysulfide-trapping mechanism and cell configuration of the lithium-sulfur (Li-S) battery using (**a**) pristine separator and (**b**) MoS_2_/graphene-coated separator.

Herein, we designed and tested a MoS_2_/graphene composite fabricated on the pristine separator through slurry coating process to use as a functional separator for Li-S batteries, as shown in Figure 1. It has been demonstrated that graphene oxide possesses outstanding sulfur adsorption capability due to its oxygen-containing functional groups [26]. The introduction of the MoS_2_/graphene-coated separator can incorporate chemisorption and physical shielding to restrain shuttle effect, leading to enhanced cycling behavior of Li-S batteries. The battery using MoS_2_/graphene-coated separator delivers a specific capacity of 1516 mAh g^−1^ at 0.2 C in the first cycle and a reversible capacity of 880 mAh g^−1^ after 100 cycles. Compared to the battery using pristine separator, the batteries using MoS_2_/graphene composite separator present significantly enhanced electrochemical properties.

## 2. Materials and Methods

### 2.1. Preparation of Flower-Like MoS_2_

In a typical procedure, 0.88 g of Na_2_Mo_4_·2H_2_O and 1.4 g of H_2_NSCNH_2_ were dissolved in 50 mL ultrapure water with vigorously stir for 50 min. Then 2 M of HCl solution was added into the mixture to adjust the pH value to 1. Subsequently, the obtained solution was transferred into a 100 mL Teflon-lined autoclave and heated to 180 °C and kept for 24 h. Lastly, the resultant MoS_2_ was rinsed with ultrapure water to neutral and then dried under vacuum overnight.

### 2.2. Preparation of MoS_2_/Graphene Composite

First of all, graphene oxide (GO) was synthesized according to Hummers’ method and diluted by distilled water to obtain a GO suspension with the concentration of 2 mg mL^−1^ [27]. Then 1 g MoS_2_ powder was dispersed in 100 mL of GO suspension and 300 mL distilled water. The mixture was stirred for 1 h to form a well-distributed MoS_2_/GO suspension, which was further dried via a spray dryer with the solution feed rate of 5 mL min^−1^ at the inlet temperature of 200 °C. Finally, the obtained black powders were collected in a cyclone collector and further reduced to MoS_2_/reduced graphene oxide (RGO) by using hydrazine hydrate.

### 2.3. Preparation of MoS_2_/Graphene-Modified Separator

To fabricate MoS_2_/graphene-modified separator, a slurry was prepared with MoS_2_/graphene composite, carbon black and poly(vinylidene fluoride) (PVDF) in N-methylpyrrolidone (NMP) with a mass ratio of 8:1:1. The homogenous slurry was then pasted on a microporous membrane (Celgard 2400) which was employed here to provide mechanical strength. After drying at 70 °C under vacuum for 12 h, the MoS_2_/graphene-modified separator was stamped into round pieces with the size of 19 mm in diameter. The mass loading of the MoS_2_/graphene was 0.80 mg cm^−2^.

### 2.4. Synthesis of S/Graphene Cathode Material

To prepare the S/graphene cathode material, 50 mL GO suspension was sealed in a 100 mL Teflon-lined autoclave and maintained at 180 °C for 12 h. After cooling to room temperature, the as-prepared RGO suspension was freeze-dried in vacuum overnight to evaporate the water solvent. Afterwards, the black precipitate was ground into powder and then RGO was obtained. Then, RGO and sublimed sulfur were mixed in the weight ratio of 75:25 using ball milling method. Finally, the mixture was then transferred into a 10 mL Teflon-lined autoclave and further maintained at 155 °C for 12 h to obtain the S/graphene composite.

### 2.5. Material Characterization

The phase components of the composites were observed with X-ray diffraction (XRD, Rigaku S4800, Rigaku Corporation, Tokyo, Japan) measurement. The X-ray photoelectron spectroscopy (XPS, K-Alpha1063, Thermo Scientific, Waltham, MA, USA) was employed to investigate the compositions of the MoS_2_/graphene-modified separator before and after cycling. The morphology of the samples was explored by transmission electron microscopy (TEM, JEM-2100F, JEOL, Tokyo, Japan) and scanning electronic microscope (SEM, Supra 55, ZEISS, SUPRA, Jena, Germany) equipped with an energy dispersive spectroscopy (EDS) system. The dynamic mechanical analysis (DMA, Q800, TA Instruments, New Castle, DE, USA) was used to measure the tensile strain of the separators.

### 2.6. Electrochemical Measurements

The cathode was prepared as follows. The sulfur/graphene composite, carbon black and PVDF (8:1:1 wt ratio) were mixed with NMP as the solvent. After grinding for 60 min, the resultant slurry was tape-cased onto aluminum foil. After drying at 60 °C under vacuum for 10 h, the obtained composite covered electrode was stamped into disks with diameter of 14 mm. The evaluation of electrochemical performance was carried out in CR2032-type coin cells and the assembly of cells was finished in an Ar-filled glovebox (MBraun, Garching, Germany). The lithium foils served as the counter electrodes. For the electrolyte, 1 M lithium bis(trifluorometha-nesulfone)imide (LiTFSI) and 0.1 M LiNO_3_ dissolved in the mixture of dioxolane (DOL) and 1,2-dimethoxyethane (DME) (1:1 ratio vol%) were employed. The MoS_2_/graphene-modified separator was inserted between the cathode and the lithium foil with the MoS_2_/graphene coated side of the separator facing the cathode. The pristine Celgard 2400 was also used for comparison. The galvanostatic cycling experiments were conducted on a battery cycler (Neware, Shenzhen, China) with a cut-off potential of 1.7–2.8 V (vs. Li^+^/Li). Cyclic voltammetry (CV) was tested on a CHI611C potentiostat (Shanghaichenhua, Shanghai, China) within the voltage range of 1.7–2.8 V with a scan rate of 0.1 mV s^−1^. Electrochemical impedance spectroscopy (EIS) was performed on a CHI611C potentiostat in the frequency range of 100 kHz–0.01 Hz.

## 3. Results and Discussion

**Figure 2:**
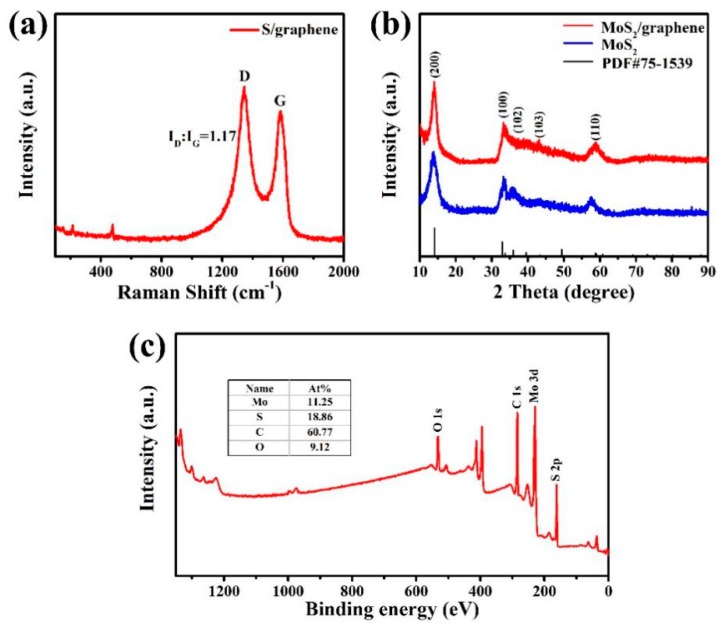
(**a**) Raman spectrum of S/graphene composite; (**b**) X-ray diffraction patterns of MoS_2_ and MoS_2_/graphene composite; (**c**) XPS spectrum of MoS_2_/graphene composite.

Raman characterization of S/graphene composite was shown in Figure 2a to confirm its composition. The Raman spectrum presents two major peaks at ca. 1344 and 1582 cm^−1^, which correspond with the D band and G band of graphene, respectively. Moreover, D band is corresponding to the disordered sp^3^ structure and G band is assigned to the sp^2^ hybridized graphitic structure. The I_D_/I_G_ value of S/graphene is 1.17, indicating a highly defective graphite structure, which is beneficial to fast electron transfer [28]. Moreover, there are three characteristic Raman peaks of sulfur at 150, 215 and 477 cm^−1^, indicating the inclusion of sulfur in the composite [29].

As presented in Figure 2b, the crystal structure of MoS_2_ and MoS_2_/graphene composite were characterized using X-ray diffraction (XRD). There are five similar diffraction peaks observed in two samples. According to the standard Powder Diffraction File (PDF) files of #75-1539, conspicuous peaks at 14.0°, 33.4°, 36.2°, 43.6° and 58.1° are indexed to (002), (100), (102), (103) and (110) planes of crystalline MoS_2_ respectively, indicating that the crystal structure of MoS_2_ was efficiently retained in the spray drying process. The peak of graphene was not obtained. 

XPS measurements were performed to investigate the surface chemical composition of the composite. The full spectrum in Figure 2c portrays the existence of Mo, S, C and O and the mass percentages of Mo, S, C and O are 11.25%, 18.86%, 60.77%, 9.12% respectively. The relatively high C content and low Mo content indicate that MoS_2_ has been coated by graphene, which was further demonstrated by SEM and TEM results.

**Figure 3:**
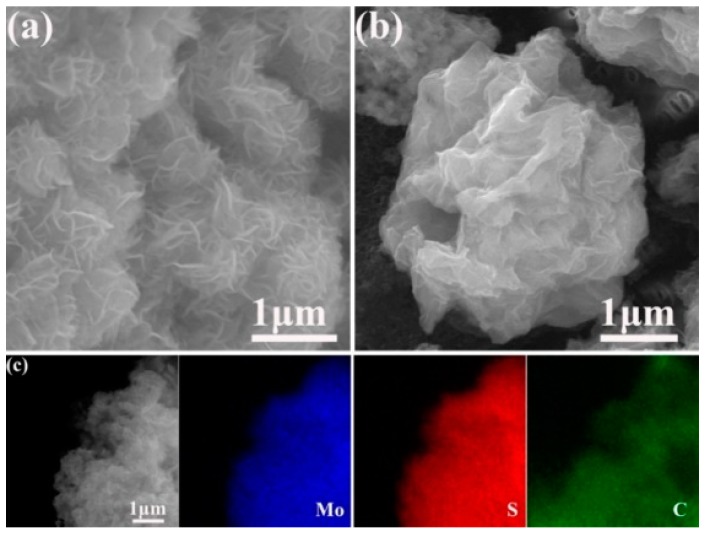
SEM images of (**a**) MoS_2_ and (**b**) MoS_2_/graphene composite; (**c**) EDS mapping of MoS_2_/graphene composite.

The morphology of as-prepared MoS_2_ and MoS_2_/graphene composite is revealed by SEM images and energy dispersive X-ray spectroscopy (EDS) mapping. As presented in Figure 3a, the as-prepared MoS_2_ exhibits a flower-like structure with the average size of around 1.1 μm in diameter. The flower-like structure consisted of nanosheets with the thickness of around 30 nm. Figure 3b reveals that the MoS_2_ nanospheres were effectively wrapped by graphene, and the three-dimensional flower-like structure was remained in MoS_2_/graphene composite. However, the diameter of the MoS_2_/graphene sphere is about 3.2 μm, which is much larger than that of MoS_2_. EDS mapping of Mo, S and C elements in Figure 3c shows uniform distribution of three elements in the whole structure, confirming that MoS_2_ and graphene are tightly bonded.

**Figure 4:**
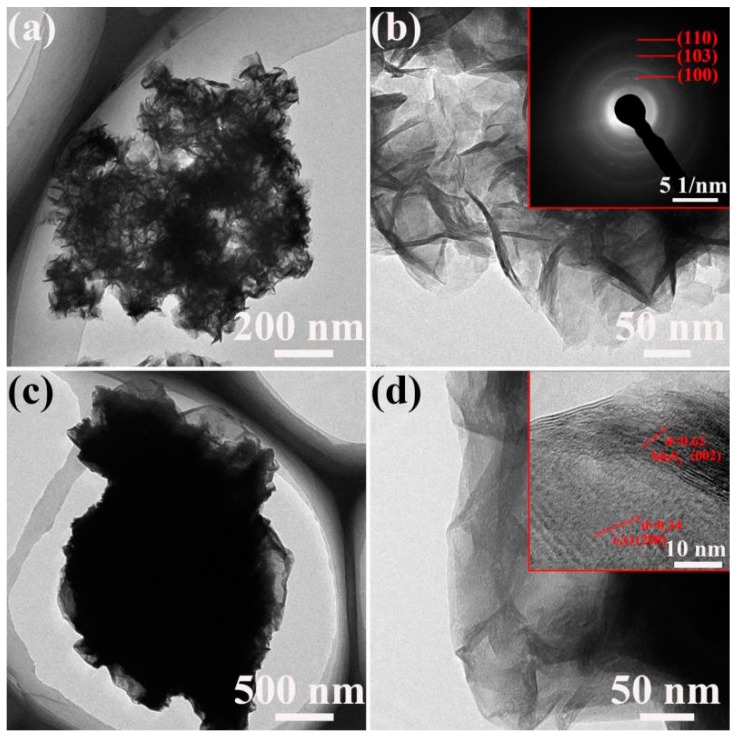
TEM images of (**a**,**b**) MoS_2_ and (**c**,**d**) MoS_2_/graphene composite.

The insight into inner structure of the samples was provided by transmission electron microscopy (TEM) analysis. Figure 4a,b shows the structure of as-prepared MoS_2_. The flower-like structure is clearly observed from the wrinkles. The inset of Figure 4b presents selected area electron diffraction (SAED) with distinct ring pattern, revealing the crystalline nature of MoS_2_. Figure 4c,d show the structure of MoS_2_/graphene composite, revealing that some nanospheres of MoS_2_ are well wrapped by thin graphene sheets. As illustrated by the HRTEM image in the inset of Figure 4d, the spacing between the adjacent fringes can be resolved to be 0.62 nm and 0.34 nm, corresponding to the (002) planar spacing of MoS_2_ and (200) planar spacing of graphene, respectively. The results indicate a strong interfacial bonding between graphene and MoS_2_, which can shorten electron/ion transport passage and supply more adsorption surfaces.

**Figure 5:**
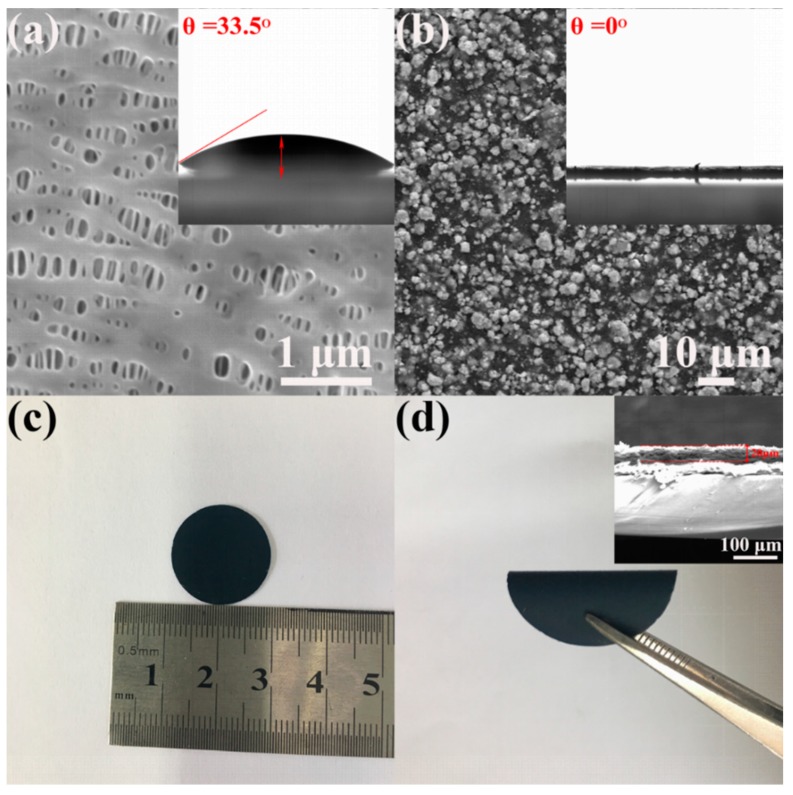
SEM images of (**a**) pristine separator; (**b**) MoS_2_/graphene-coated separator (The insets are the contact angle of electrolyte on the surface of each separator); (**c**,**d**) Photographs of the MoS_2_/graphene-coated separator (inset is the image of MoS_2_/graphene-coated separator section).

The surface morphologies of the pristine and MoS_2_/graphene-coated separators are investigated by SEM. As presented in Figure 5a, the surface of pristine separator is flat and nanoporous. The size of pores was around 100 nm in diameter, which can effectively guarantee the rapid transfer of ions and achieve the diffusion of polysulfides to anode [30,31]. Moreover, the wetting ability of the separators was tested with contact angle measurement. The inset in Figure 5a exhibits that the contact angle of the pristine separator is 33.5°. Figure 5b shows the surface morphology of the MoS_2_/graphene-coated separator. Uniform MoS_2_/graphene spheres with the average size of 3.6 μm in diameter were observed. The contact angle of the MoS_2_/graphene-coated separator is 0° as shown in the inset in Figure 5b, suggesting the excellent wetting properties of the composite separator. The images of MoS_2_/graphene-coated separator with tiled and folded state are exhibited in Figure 5c,d respectively. The separator shows a preeminent flexibility, which can guarantee the close contact between the surface of the sulfur cathode and the separator. The cross-section of the MoS_2_/graphene-coated separator (inset of Figure 5d) reveals good adhesion of MoS_2_/graphene composite to the surface of the pristine separator, and composite layer shows a thickness of around 20 μm.

**Figure 6:**
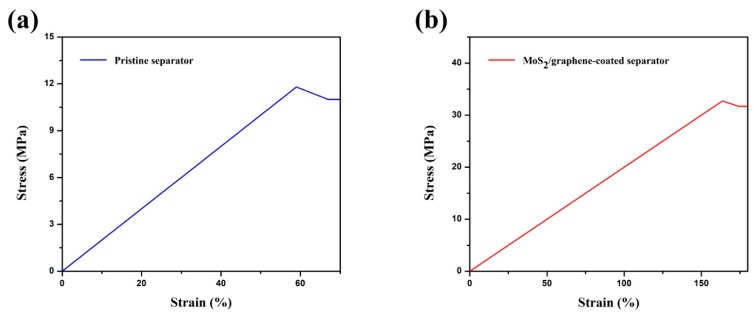
The mechanical properties of (**a**) pristine separator and (**b**) MoS_2_/graphene-coated separator.

The tensile property was measured to the mechanical properties of pristine separator and MoS_2_/graphene-coated separator. As shown in Figure 6, the elasticity modulus of pristine separator and MoS_2_/graphene-coated separator are calculated from curves to be 0.202 and 0.203, respectively. It is evident that the coating of MoS_2_/graphene on the surface of pristine separator does not affect the elasticity of the pristine separator. Notably, the tensile strength of MoS_2_/graphene-coated separator is 33 MPa, which is higher than that of the pristine separator, indicating the excellent elongation and mechanical properties. This can be attributed to the high flexibility property of the MoS_2_/graphene membrane.

**Figure 7:**
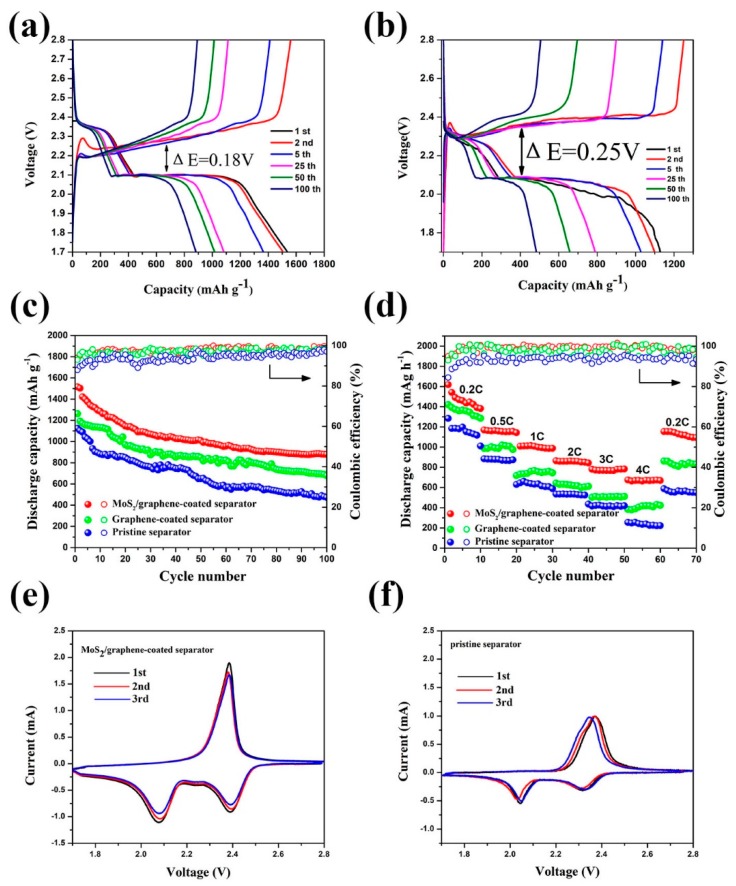
The discharge/charge voltage profiles of Li-S batteries with (**a**) MoS_2_/graphene-coated separator and (**b**) pristine separator at 0.2 C; (**c**) The cycling performance and (**d**) rate capability of Li-S batteries with MoS_2_/graphene-coated, graphene-coated and pristine separator; The cyclic voltammetry (CV) curves of the Li-S batteries with (**e**) MoS_2_/graphene-coated separator and (**f**) pristine separator.

The electrochemical measurements were performed to clarify the benefit of the MoS_2_/graphene-coated separator in enhancing the performance of Li-S batteries. The representative discharge/charge curves of the batteries with MoS_2_/graphene-coated separator and pristine separator between 1.7 and 2.8 V at 0.2 C are presented in Figure 7a,b. The profiles show two voltage plateaus upon discharging and one voltage plateau upon charging, which is consistent with the previous reports [32,33]. One can see that the discharge/charge curves of the batteries with MoS_2_/graphene-modified separator exhibit more steady voltage platforms and smaller capacity loss compared to the one with pristine separator. Meanwhile, ΔE for cells with pristine separator, which is 0.25 V, is larger than that for cells with MoS_2_/graphene-coated separator, which is 0.18 V. These results demonstrate that the MoS_2_/graphene-coated separator relieves the redox polarization in the batteries. The lowering of potential hysteresis can be attributed to the abundant electron/ions transport channels and huge adsorption surfaces provided by the strong interfacial bonding between MoS_2_ and graphene, which can effectively promote the transmission of electrons and ions to accelerate the kinetics processes of the electrochemical reactions [34].

Figure 7c shows the cycling capability of the batteries with different separators at 0.2 C. The batteries with the pristine separator exhibit the first discharge capacity of 1125 mAh g^−1^ and the 100th discharge capacity of 473 mAh g^−1^, which indicate a rapid decay of discharge capacity. The batteries with graphene-coated separator show slightly improvement on discharge capacity and cycling performance with the first discharge capacity of 1363 mAh g^−1^ and the 100th discharge capacity of 706 mAh g^−1^, resulting in a capacity retention of 51%. These improvements can be due to the high conductivity of the graphene-coated separator, which can effectively shorten the transport passage of electrons and ions. Notably, the batteries using the MoS_2_/graphene-coated separator possess much better cycling performance. The first discharge capacity increased to 1516 mAh g^−1^, indicating that the lithium polysulfides solved in the electrolyte is successfully intercepted by the MoS_2_/graphene-modified separator in the initial discharge process. After 100 cycles, the batteries using MoS_2_/graphene-coated separator maintain a capacity of over 880 mAh g^−1^. Moreover, the batteries with MoS_2_/graphene-coated separator also exhibit a higher coulombic efficiency of about 98.7%. 

The rate capability comparison of the batteries using different separators was also investigated. As shown in Figure 7d, the battery using the pristine separator exhibits a poor performance, which delivers 1197, 878, 632, 533, 420, 235 mAh g^−1^ at 0.2 C, 0.5 C, 1 C, 2 C, 3 C and 4 C, respectively. Moreover, the rate capability of the battery using the graphene-coated separator shows a modified performance due to the excellent conductivity of graphene-coated separator, which delivers 1391, 992, 732, 631, 542, 395 mAh g^−1^ at 0.2 C, 0.5 C, 1 C, 2 C, 3 C and 4 C, respectively. By contrast, the battery using the MoS_2_/graphene-modified separator possesses much enhanced rate capability with the initial discharge capacity of 1618 mAh g^−1^ at 0.2 C, which was much higher than batteries with graphene-coated separator and pristine separator. After that, the capacity gradually decreases and stabilizes at around 1382 mAh g^−1^. Furthermore, the battery with MoS_2_/graphene-modified separator delivers high reversible capacities of 1163, 1009, 860, 770 and 671 mAh g^−1^ at the rate 0.5 C, 1 C, 2 C, 3 C and 4 C, respectively. Notably, after the rate reverted to 0.2 C, a high capacity of 1130 mAh g^−1^ is recovered. The significantly enhanced electrochemical performance can be ascribed to the three-dimensional flower-like MoS_2_/graphene composite, which are beneficial for fast transport of ion/electronic as well as increase the liquid electrolyte storage capacity. 

Figure 7e exhibits the initial three cyclic voltammetry (CV) profiles of batteries using MoS_2_/graphene-coated separator. Two reduction peaks at 2.1 and 2.4 V are detected, which can be ascribed to the formation of sulfur to long chain polysulfides and then to Li_2_S/Li_2_S_2_. The only oxidation peak at 2.4 V is corresponding to the reverse conversion of Li_2_S/Li_2_S_2_ into the polysulfides as well as elemental sulfur. The CV profiles in the subsequent scans show no distinct change of the peak position and intensity, suggesting an outstanding electrochemical stability of the batteries. However, for CV profiles of batteries with the pristine separator, the intensity of the peaks shows obvious decrease, indicating larger polarization in the batteries, as shown in Figure 7f. Moreover, the loop area of the battery with MoS_2_/graphene-modified separator is larger than that of the one with the pristine separator, suggesting the high utilization of sulfur for energy storage using the MoS_2_/graphene-coated separator [35].

**Figure 8:**
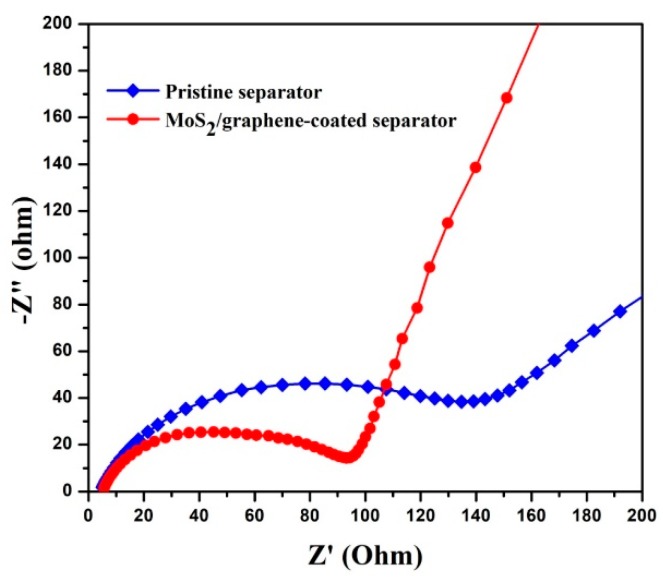
The electrochemical impedance spectra of Li-S batteries using pristine separator and MoS_2_/graphene-coated separator.

To study the kinetics process, EIS measurements for the batteries with pristine separator and MoS_2_/graphene-coated separator were performed. As presented in Figure 8, both Nyquist plots are composed with a semicircle in high frequency region and an inclined line in the low-frequency region, which are in connection with the charge-transfer resistance and Li^+^ diffusion, respectively. Notably, the batteries with MoS_2_/graphene-coated separator shows much lower charge transfer resistance than the battery with the pristine separator (89 Ω versus 135 Ω). The lower resistance of the batteries with MoS_2_/graphene-coated separator results from decreased insulating materials deposited on the surface of the electrode, leading to an excellent cycling stability.

**Figure 9:**
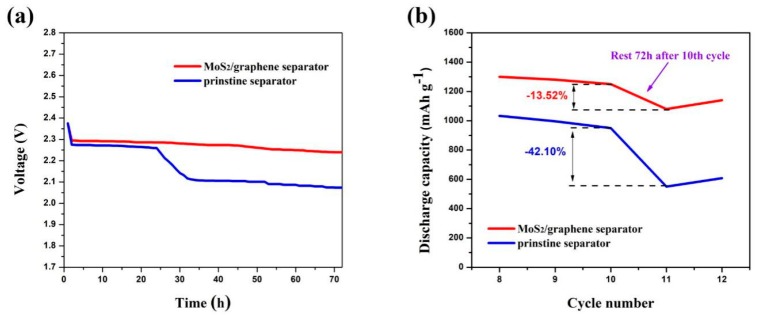
Self-discharge behavior of the electrodes with the MoS_2_/graphene-coated separator and the pristine separator. The cells were rested for 72 h after the 10th cycle: (**a**) open-circuit voltage profiles during the rest time; (**b**) cycling performance of the electrodes.

Since the self-discharge is one of the major defects of Li-S batteries, the self-discharge behavior of the electrodes with the MoS_2_/graphene-coated separator and the pristine separator were characterized. The batteries were rested for 72 h after the 10th cycle at 0.2 C. Figure 9a shows the open-circuit voltage profiles of both cells. One can see that the voltage for the battery with the pristine separator obviously dropped from 2.26 to 2.11 V during the rest time, indicating the significant reduction of the polysulfides from high-order to low-order. However, the voltage of the battery using the MoS_2_/graphene-modified separator shows a small attenuation from 2.3 to 2.26 V during the rest time, indicating the effective suppression of the self-discharge by the composite separator. Figure 9b shows the cycling performance of the electrodes after the 72 h rest. The original capacity of the battery using a pristine separator was significantly reduced by 42.1%. In contrast, the battery using a MoS_2_/graphene-coated separator exhibited a decline rate of 13.52%.

**Figure 10:**
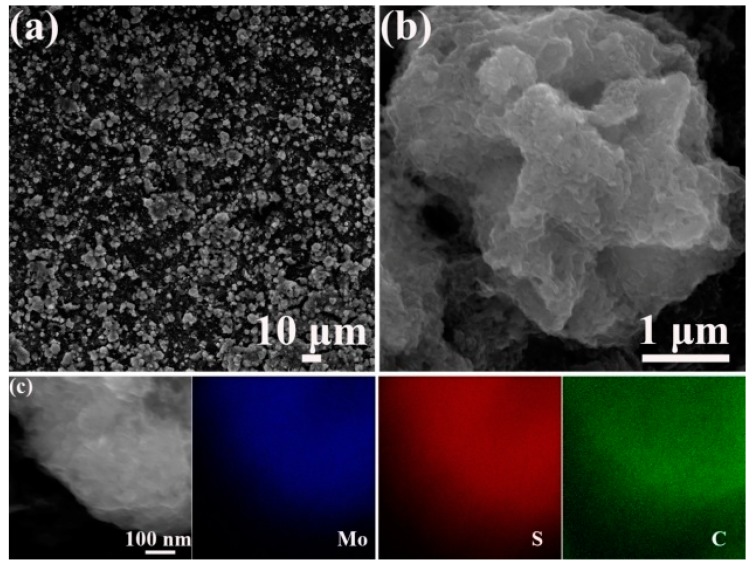
(**a**,**b**) SEM images of the MoS_2_/graphene-coated separator after 100 cycles at 0.2 C and (**c**) the elemental mapping of Mo, S and C.

The morphology of the MoS_2_/graphene-coated separator after 100 cycles at 0.2 C is detected and the results are exhibited in Figure 10a,b. Compared with the data before cycling in Figure 3, the surface was slightly damaged, and the MoS_2_/graphene composite still remain uniform distribution on the surface, indicating that the coating is flexible and tightly coated to avoid dropping out from the separator during cycles. No obvious cluster was observed and the diameter of the MoS_2_/graphene sphere is about 4 μm. The corresponding EDS elemental maps (Figure 10c) further reveal that the Mo, S and C are distributed homogenously across the whole structure after cycles, suggesting that the composite coating acted as a filter for absorbing the dissolved polysulfides.

**Figure 11:**
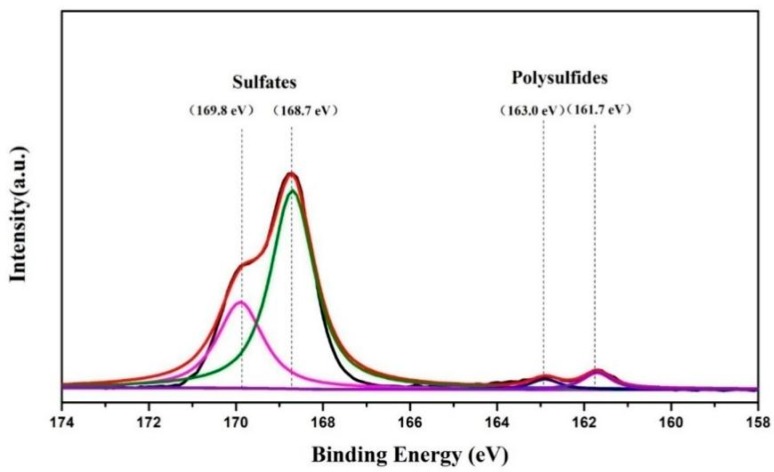
Ex-situ S 2p XPS spectra of the cycled MoS_2_/graphene-coated separator.

XPS S 2p spectrum of the MoS_2_/graphene-coated separator after 100 cycles at 0.2 C was presented in Figure 11 to verify the interaction between polysulfides and the MoS_2_/graphene-modified separator. Here the peaks between 169.8 and 168.7 eV are assigned to the sulfates, which can be attributed to brief air exposure of the sample in the XPS loading process. Furthermore, the peaks at 163.0 and 161.7 eV prove the existence of polysulfides in the MoS_2_/graphene-modified separator, consistent with the previous SEM results. Therefore, we believe that the migrating polysulfide species can diffuse through the MoS_2_/graphene-modified separator and be effectively captured during cycling.

## 4. Conclusions

In summary, a multi-functional separator modified by MoS_2_/graphene composite is synthesized on the routine separator via slurry coating process to substantially enhance the electrochemical capability of Li-S battery. The MoS_2_/graphene-coated separator serves as a high efficiency ionic sieve that can block the transportation of the polysulfide anions and effectively decreases the shuttle effect. In addition, the introduction of MoS_2_/graphene-coated separator possessed higher sulfur utilization as a result of the recycling of entrapped active materials. These advantages can significantly improve the specific capacity and cycling stability of battery, with an initial discharge capacity of 1516 mAh g^−1^ and a higher reversible capacity of 880 mAh g^−1^ with coulombic efficiency of 98.7% after 100 cycles at 0.2 C. Therefore, the separator modification by MoS_2_/graphene composite will provide an effective method to suppress the shuttling phenomenon and enhance the electrochemical capability of Li-S batteries.
